# Word usage as measured by parental checklists and language samples: trends, comparisons, and implications

**DOI:** 10.3389/fpsyg.2023.1214518

**Published:** 2023-08-03

**Authors:** Daniela Gatt, Liberato Camilleri, Chloe Grech

**Affiliations:** ^1^Department of Communication Therapy, Faculty of Health Sciences, University of Malta, Msida, Malta; ^2^Department of Statistics and Operations Research, Faculty of Science, University of Malta, Msida, Malta

**Keywords:** vocabulary checklist, parental report, language sampling, word frequency, vocabulary acquisition, bilingual acquisition

## Abstract

**Background:**

Although parental checklists are well-known for their potential in indexing young children’s lexicon size, they can also be used to track children’s acquisition of individual words. Word-level data can be used to identify the checklist words most and least commonly employed across groups of children. Like parent-completed vocabulary checklists, samples of spontaneous language use collected from multiple children can also generate measures of word commonality, concerned with the numbers of children producing individual words. To our knowledge, comparisons of word usage as determined by parental checklist and language sample data obtained in parallel from the same children have not been carried out. Also scarce in the empirical literature are item-level analyses of early bilingual lexicons that explore word usage across two emerging languages. The present study aimed to contribute toward bridging both gaps through the analysis of data generated by a bilingual Maltese-English adaptation of the vocabulary checklist of the MacArthur Communicative Development Inventories: Words and Sentences (CDI: WS) and spontaneous language samples for the same children. An additional objective was to derive implications for revising the current version of the vocabulary checklist, in preparation for its eventual standardization.

**Materials and methods:**

For 44 Maltese children aged 12, 18, 24, and 30 months, the words reported by their main caregivers on the vocabulary checklist were identified, along with their respective semantic categories. For the same children, 20-min language samples obtained during free play with the caregiver were transcribed orthographically. Words identified through parental report and language sampling were analyzed for commonality, i.e., the number of children producing each word.

**Results:**

Comparison of the word usage patterns obtained through both methods indicated differences in the words most commonly sampled and those most commonly reported, particularly in relation to grammatical categories. Notwithstanding these differences, positive and significant correlations emerged when considering all grammatical categories and languages across commonality levels.

**Discussion:**

The commonality scores based on parental checklist data have implications for reconsidering the length and language balance of the Maltese-English adaptation of the CDI: WS vocabulary checklist. Sampled word usage patterns can contribute additional objectivity in updating the reporting instrument in preparation for its eventual standardization.

## 1. Introduction

There is a wealth of evidence in favor of using parent report tools to measure young children’s language skills for research and clinical purposes. When parents and primary caregivers are asked to describe their children’s emergent language through interviews, questionnaires and checklists, they are known to impart reliable and valid information. This is because, typically, primary caregivers are attentive to their children’s early language milestones, monitoring their emergent skills closely across daily settings (Fenson et al., 1994). In particular, parental report enables the collection of comprehensive vocabulary data from extensive samples of children, enhancing the recognition of universal trends and natural variability in language acquisition through a relatively undemanding method (Frank et al., 2017). Reported vocabulary data also contribute to a better understanding of the developmental trajectories of individual words (Fenson et al., 1994) and how these compare across different languages (Frank et al., 2021).

### 1.1. The MacArthur-Bates Communicative Development Inventories

The MacArthur-Bates Communicative Development Inventories (CDIs) are among the parent-report instruments most widely used to measure children’s early language skills, including vocabulary. Adaptations of the original U.S. English version span several languages (see Dale and Penfold, 2011). Importantly, CDI vocabulary measures are not intended as an exhaustive inventory of the words known by the child, for comparison with measures generated by other tools (Frank et al., 2021). Rather, they function as an index of children’s lexical abilities relative to their peers’, measured through the same instrument (Fenson et al., 1994).

Although vocabulary checklists tap strategically into parents’ familiarity with their children’s early lexicons, they are also subject to reporting biases. Parental estimates of children’s early vocabulary and emergent grammar skills may be inaccurate, particularly if lower income and educational levels are present (e.g., Roberts et al., 1999; Feldman et al., 2000). Socioeconomic variables are less consistently associated with early language difficulties than neurobiological factors (Rescorla and Dale, 2013), suggesting that lower parent-based scores among disadvantaged groups may be attributed more directly to incomplete reporting than to the impact of unfavorable environmental conditions. Besides, details reported by parents would have been filtered through their own subjective perceptions (Stiles, 1994). In fact, vocabulary checklists attempt to optimize parents’ reporting ability by prompting them to record their children’s current and newly emerging lexical skills through a recognition format (Dale, 1996). The addition of words recollected by parents from memory may be relevant during the earlier stages of instrument design (e.g., Fenson et al., 1994). Reliance on a recognition format also means that the extensiveness of parent-reported data is regulated by the specific reporting opportunities, that is, the words available on the checklist for ticking. Nonetheless, the wealth of in-depth and dependable vocabulary data yielded by parental report goes a long way in mitigating its methodological bias. In fact, the CDI vocabulary checklists have an impressive track record of eliciting reliable and valid measures of children’s expressive lexicon size (e.g., Galeote et al., 2016; Frank et al., 2021; de Anda et al., 2022).

### 1.2. Comparisons between parental report and language sample measures

The validation of newly-developed parent report instruments requires comparability to direct assessment measures. Given the scarcity of norm-referenced tests for young children, concurrent measures for establishing validity are often obtained through language sampling and informal structured assessment. Parental estimates of children’s vocabularies have been compared to the lexical skills emerging spontaneously during naturalistic observation, or elicited through structured testing, with positive and significant correlations often resulting (see Fenson et al. (2007) for a review). Moderate to high correlations with sampled vocabulary measures have also been reported for CDI adaptations to numerous languages representing a range of language families, e.g., Spanish (Jackson-Maldonado et al., 1993); Danish (Bleses et al., 2008); Kigiriama and Kiswahili (Alcock et al., 2015), as well as bilingual adaptations for children exposed to specific language pairs (see Gatt et al. (2014) for Maltese-English; O’Toole and Fletcher (2010) for Irish-English), as well as language adaptations for children with specific disorders (see, e.g., Galeote et al. (2016) for the Spanish adaptation for children with Down Syndrome), substantiating the concurrent validity of CDI-based vocabulary measures.

Comparisons of parent-reported and sampled vocabulary scores have also served the purpose of establishing whether the presence of a noun bias in children’s early lexicons is subject to methodological influences. Substantial cross-linguistic evidence points toward a general mechanism in vocabulary composition, whereby young children’s expressive vocabularies start off with a predominance of social words (e.g., sound effects and routine words) which gives way to nouns, followed by a subsequent emphasis on predicates, comprising main verbs and adjectives, and eventually culminates in function words. Earlier findings in the field drew on the original CDI (Bates et al., 1994) and its adaptations to other languages (e.g., Caselli et al. (1999) for Italian). Although a noun bias has also been identified through spontaneous language sampling (see Bassano (2000) for French; Kauschke and Hofmeister (2002) for German), conflicting evidence (e.g., Tardif et al., 1999) prompted consideration of methodological factors potentially impacting on children’s vocabulary composition. A small body of research has therefore employed a combination of caregiver report and sample measures to minimize methodological bias, while keeping language constant. For example, when employing reported and sampled vocabulary measures in parallel, Salerni et al. (2007) consistently identified a predominance of nouns in Italian’s children’s 200- and 500-word vocabularies. However, significant differences across methods emerged in grammatical category proportions.

Such findings underscore the fact that the choice of method for documenting early vocabulary skills may influence the measures obtained. However, the parameters of this methodological distinction may appear ambiguous, particularly since the empirical literature shows parental report and language sampling to concur time and again in their measurement of expressive lexicon size. Yet, they are intrinsically different. Checklist measures of vocabulary span various daily settings in which parents observe their children’s available language skills. In contrast, sampled vocabulary production draws on a limited window in which the child’s compliance, interlocutor’s input and context of interaction bear directly on the amount and representativeness of data obtained (Bates et al., 1988; Frank et al., 2021). Observed language behaviors have been fittingly described as ‘sporadic’ (Fenson et al., 1994: 11).

Despite differences in absolute scores, reported and sampled vocabulary size for the same children tend to rank similarly. However, the occurrence of specific words and their frequency of production are known to be highly sensitive to sampling parameters, such as the toys employed during free play (Frank et al., 2021). In contrast, words reported by parents draw on the child’s participation in various interactional exchanges and do not incorporate information on frequency of occurrence. This difference was aptly synthesized by Bates et al. (1988), who contrasted vocabulary checklists’ potential to identify the words children *know* with language samples’ potential to capture the words children *use*. In terms of actual word usage, the two methods are therefore expected to be discordant, suggesting that differences between methods at the item level may be more pronounced than for composite counts of words. This could partly explain the marked absence of comparisons between checklist and sample word-level data from the research literature.

The purpose of the present study was twofold. First, it identified the more commonly produced words identified through each method for a single cohort and investigated the extent to which these reported and sampled words overlapped. Second, it sought to derive guidelines for a subsequent iteration of the Maltese-English vocabulary checklist adaptation, in preparation for norming. The CDI Advisory Board recommends that CDI adaptations are piloted extensively, with detailed item-level analyses of checklist data and language sampling being requisites for arriving at a final set of words that is amenable to norming. A careful, data-driven, approach is particularly critical to the development of parent report instruments intended to measure early bilingual vocabularies, since extensive individual variability stemming from language exposure variables is expected (Weisleder et al., 2022). Revising the current checklist in light of this study’s findings would be a crucial step toward eventually standardizing the Maltese-English CDI adaptation. A new revision of the Maltese-English vocabulary checklist adaptation would comprise the fourth iterative cycle of the instrument.

The methodological issues addressed by this study also have theoretical ramifications that stem from its focus on an under-researched language pair. Recent years have seen more publications on Maltese children’s bilingual acquisition of Maltese and English in prominent language acquisition journals than in the past (Kidd and Garcia, 2022). However, documentary evidence is still largely lacking, despite the fact that Maltese and English are two languages with highly dissimilar typologies, making for more valuable comparisons across them (Slobin and Bowerman, 2007). Moreover, the normative bilingual context in which these two languages are acquired (Gatt and Dodd, 2019) adds to the relevance of documenting Maltese children’s bilingual acquisition in detail, particularly since the study of normative bilingualism holds immense theoretical potential (Montanari and Nicoladis, 2016).

The nature and scope of word usage data, from the methodological perspective of parental report and language sampling, is reviewed in the next section. In the present text, we use the term ‘word usage’ to refer to the occurrence of individual words in vocabulary data, so that our primary focus is on item-level trends in children’s expressive vocabularies rather than on aggregated scores of vocabulary size.

### 1.3. Word-level vocabulary checklist measures

Vocabulary checklists can never claim to include all the words that young children understand and/or produce as their language skills emerge. The diversity of words that young children accumulate through interactions in specific language-learning environments, together with the rapidity with which their vocabularies grow, imply that beyond the stage of children’s first words, an exhaustive vocabulary checklist is barely conceivable. Its sheer length would also make it unwieldy and daunting to complete. Vocabulary checklists therefore seek to present parents, or primary caregivers, with a sample of words that realistically represent children’s lexical repertoires (Fenson et al., 1994). Arriving at a set of words that best characterizes typically-developing children’s varying levels of typical lexical development is one objective of item-level vocabulary measures. For example, the current version of the U.S. English CDI vocabulary checklists was developed from several iterations based on data collected through parental questionnaires that preceded the CDIs (Fenson et al., 1994). Scrutiny of these data shed light on the psychometric properties of the instruments. In particular, information on the rate and pattern of growth shown by individual words delineate their sensitivity to developmental change. Usage of individual words reported across children plays a role in informing decisions on which items to discard and retain, so that composite scores across all words can then reflect vocabulary development tendencies (Fenson et al., 1994). Importantly, checklist versions in other languages are emphasized to be adaptations, rather than translations. The original list of words should be assessed for cultural and linguistic relevance to the population of interest. The MacArthur-Bates Advisory Board encourages a similar distribution of words across difficulty levels and grammatical categories in CDI adaptations as in the original (Frank et al., 2021). Studies evaluating the psychometric properties of newly-adapted instruments may therefore resort to analyses of item-level performance. For example, Weber et al. (2018) examined children’s responses to individual items on the Wolof adaptation of the CDI:WS short form, to ascertain that the set of words captured varying levels of vocabulary ability for Wolof-learning children in the target age range.

### 1.4. Word-level language sample measures

In the analysis of child language samples, two expressive vocabulary measures that feature often are type and token counts, representing the number of different words and total number of words produced, respectively. Although their computation involves scrutiny of individual words produced to distinguish the unique and recurring ones, the resulting measures are broad-based rather than focused on individual items. A body of research, however, has gone beyond the identification and counting of early words emerging in naturalistic contexts, zooming in on the occurrence of each word in terms of commonality and frequency. In a landmark study by Beukelman et al. (1989), every word occurring in classroom language samples obtained from six typically-developing children was examined for commonality, or consistency of use across the group (i.e., the number of children employing it), as well as frequency of use by each child and across participants. These measures, obtained from 3- and 4-year-olds, were intended to guide the choice of vocabulary for non-verbal classmates using augmentative and alternative means of communication (AAC). The most commonly used words, referred to as core vocabulary, were also those occurring most frequently in the samples. In contrast, fringe vocabulary consisted of words showing limited usage. Fringe word repertoires are much larger than core vocabularies, reflecting personalized interests and routines (Trembath et al., 2007). Studies measuring word usage by children aged 3 and younger are very limited. This could be partly due to the delicate task of assigning word status to early productions that are partly or largely unintelligible (see Vihman and McCune (1994) for a detailed discussion of early word identification). Also conspicuous is the paucity of sampled word usage investigations in bilingual contexts. To our knowledge, only Robillard et al. (2014) have addressed bilingual children’s word usage, with a sub-group of their school-aged participants being French-English speakers. Moreover, lengthy and labor-intensive transcription procedures likely explain the compromise between number of participants and sampling duration required in study designs. For example, Banajee et al. (2003) analyzed word usage for a sample of 50 typically-developing English-speaking children aged 24–36 months. Analysis drew on the first 150 utterances produced during two daily activities in nursery and daycare settings over three days. Trembath et al.’s (2007) study of word usage focused on a sample of six typically-developing Australian children including 3-year-olds (range = 3–5 years). For each child, analysis was based on a sample of 3,000 words collected in their preschool classroom. In a narrative review of research literature in the field, Laubscher and Light (2020) flagged the considerable variation across published word lists, attributing this to differences across studies in methods, contexts of sampling and criteria for defining words. Beyond these differences, function words are consistently prominent in core word lists, with nouns featuring rarely (see Beukelman et al., 1989; Banajee et al., 2003; Trembath et al., 2007). More recently, Frick Semmler et al. (2023) examined seven published core vocabulary lists, five of which also featured in Laubscher and Light’s (2020) review. While highlighting the general predominance of verbs, findings also revealed that none of the listed words appeared before the age of 25 months in typically-developing children.

### 1.5. Comparisons between reported and sampled measures at the word-level

It is noteworthy that item-level comparisons of expressive vocabularies as documented by caregiver report and direct assessment methods have rarely been reported. Among these few investigations is Dale’s (1991) comparison between words elicited from English-speaking 24-month-olds on a standardized picture naming test and CDI items reported by their parents. On the items common to both instruments, average agreement was 72.5%, supporting the CDI’s validity. Most mismatches resulted from words reported by parents not emerging on direct assessment, with factors such as children’s compliance considered as likely contributors. Ring and Fenson (2000) compared 40 toddlers’ CDI expressive vocabulary scores to parents’ estimates of the children’s picture naming skills and their actual naming performance. The purposely-designed picture booklet contained images of 35 words available for reporting on the CDI. Mean naming judgment scores were higher than actual naming performance for all but three items having moderate to difficult levels. Positive and significant correlations between CDI-based scores, parental naming estimates and child naming scores, even when checklist scores were based on a random sample of items not represented on the naming task, were taken as support for checklist validity. Gatt et al. (2014) investigated the correspondence between individual checklist items reported by parents of Maltese children aged 12–30 months and words produced by the children themselves on an informal picture naming task, reporting a percentage agreement in the range of 78–84%. Beyond the matching of checklist words and lexical items elicited through structured vocabulary tasks, there does not seem to be research comparing the individual words reported by parents to those sampled naturalistically. In the study reported here, we address this gap in the research literature.

Prior to evaluating trends in the usage of reported and sampled words, the parameters of the comparison need to be established. A fundamental consideration is the divergences between parental report and language sampling in the terminology adopted and the nature of the data yielded. First, checklist and sample data construe frequency of word usage differently. Word frequencies derived from CDI data specify the numbers of children reported to use each checklist entry (see Fenson et al., 1994; Frank et al., 2021). In sample data, frequency quantifies word occurrences in a snapshot of naturalistic language use, at individual or group level. The number of times a word occurs in sample data collected from different individuals may be referred to as ‘composite frequency’ (e.g., Trembath et al., 2007). Commonality, defined as the number of participants using a particular word during sampling (e.g., Banajee et al., 2003), is akin to word frequencies derived from checklist data. Further, the frequency of production of individual words generated by sampled vocabulary data cannot be gleaned from completed checklists. To avoid confusion, in our measures and results we use the term ‘commonality’ to refer to the number of children using a specific word in both checklist and sample data; we take ‘frequency’ to denote the number of times an item occurs in participant samples considered collectively. Second, sampled ‘core’ word data and parent-reported vocabulary inventories are also intrinsically different in layout and purpose. Core vocabularies are relatively short, functioning as “a framework for functional language use” (Banajee et al., 2003, p. 68) and remaining consistent across settings and individuals. In contrast, parent-based vocabularies bring together a range of words commonly known by young, typically-developing children. This is because they draw on the contents of the CDI vocabulary checklists, which contain words most commonly understood and produced by typically-developing children within the specified age bracket. Thus, a child’s lexicon size can be estimated by the parent and, depending on the availability of norming data, compared to standardized measures obtained for the instrument. Interestingly, Laubscher and Light (2020) point out that checklist items are very different from the words ranking highly in language sample corpora, according to their review of various core vocabulary lists in the empirical literature. These lists consistently feature a preponderance of function words, along with a scarcity of nouns and social words, that together represent only around one fifth of the items listed in the CDI vocabulary inventories. Laubscher and Light’s (2020) comparative tabulations revealed that words available for reporting in CDI instruments coincided minimally with sampled core nouns, while pronouns, question words, prepositions and other function words on core lists show the highest percentage overlap. Limited overlap between sampled core words and CDI checklist items was also reported by Frick Semmler et al. (2023). Both studies attributed this disparity to CDI checklists capturing individualized components of young children’s typical vocabularies, which core vocabulary lists are unable to detect due to sampling constraints.

### 1.6. Research questions

To our knowledge, there have been no studies that compared word usage as documented through parental report and language sampling for the same children. The present study addresses this evidence gap. It investigates word usage in a cohort of 12-30-month-olds predominantly exposed to Maltese. Measures obtained in a naturalistic setting were compared to those derived from caregiver report, which was based on a Maltese-English adaptation of the CDI: WS vocabulary checklist. The research questions addressed are the following:

1. Which words were most commonly reported by caregivers for Maltese children aged 12, 18, 24, and 30 months, in terms of grammatical category and language?
2. Which words were most commonly used by the same children spontaneously during free play with their caregivers, in relation to grammatical category and language? Which words were most frequently used?
3. How did the most commonly reported words compare to the most commonly and the most frequently sampled words, for the same children? Were there similarities when word class and language were considered?

## 2. Materials and methods

### 2.1. Participants

The participants were 44 typically-developing Maltese children aged 12, 18, 24, and 30 months. Each age group consisted of approximately equal numbers of boys and girls (12 months: 6 boys, 5 girls; 18 months: 5 boys, 7 girls; 24 months: 5 boys, 6 girls; 30 months: 5 boys, 5 girls). The main caregivers of all participants were mothers, except for one 30-month-old boy who was mostly cared for by his grandmother. While all the children’s parents had a secondary level of education, 17 mothers and 16 fathers had pursued their studies to post-secondary level. Eleven parents were in possession of a university degree, with one mother and one father also having a postgraduate qualification. Seven children were randomly selected from the National Register of Births in Malta. The remaining children were identified through snowball sampling. None of the participants manifested features that clearly impaired their language development at the time of data collection. In the absence of norms for early language acquisition based on Maltese children, participants were judged to be developing typically by the speech-language pathologist collecting the data, the first author. Data from all potential participants were collected by the same person, so clinical judgment was applied uniformly. No significant medical conditions were reported for any of the children. Preterm birth at 32 and 34 weeks (*N* = 2), occurrence of middle ear infections (*N* = 10) and the presence of speech or language difficulties in an older sibling (*N* = 2) (none reported in the parents) were not considered as exclusionary criteria. Since the two participants born prematurely were healthy preterm infants, they were likely to have better language outcomes than preterm infants with identified medical conditions (see Loeb et al., 2020). Roberts et al.’s (2004) meta-analysis identified very small associations, if at all, between a history of otitis media in early childhood and later language outcomes. Moreover, preterm birth and middle ear infections do not feature in models of strongly weighted risk factors for later language disorder (e.g., Ellis and Thal, 2008; Zambrana et al., 2014; Bishop et al., 2016). Fisher’s (2017) systematic review and meta-analysis reported that the presence of a speech or language disorder, or learning disability, in parents and/or siblings, was not a significant predictor of expressive language outcomes in late talkers. In view of these research findings, children were not excluded from the study on the basis of preterm birth, middle ear infections or speech or language difficulties in older siblings, since these factors did not appear to impact language skills at the time of data collection and were not necessarily predictive of later language difficulties. The study was approved by the University of Malta Research Ethics Committee. Primary caregivers gave informed proxy consent for their children’s participation in the study and consented to their own involvement.

Each child was exposed primarily to Maltese within the home context. In Malta, both Maltese and English carry the status of official languages, with bilingualism being widespread. Maltese, the national language, is essentially Semitic in origin but incorporates Romance and English borrowing (Hoberman, 2007). Typologically, Maltese is very different from English, a Germanic language. Among the characteristics of the Maltese language are its rich inflectional and derivational morphology, its optional subject forms, made possible by the person, number and gender inflections coded on the verb, and its free word order (Borg and Azzopardi-Alexander, 1997). Free and suffixed pronouns are marked for first, second and third person, with singular and plural distinctions also coded for each person. Pronominal suffixes attached to nouns mark possession, to verbs, where they mark direct and indirect objects, and to prepositions as their objects (Borg and Azzopardi-Alexander, 1997; Hoberman, 2007).

Maltese is the dominant language of most Maltese individuals (National Statistics Office, 2007; Vella, 2013 National Council for the Maltese Language, 2021). Since Maltese and English exist in close proximity with each other, they exert a degree of cross-linguistic influence on each other. The variety of English spoken in Malta is often referred to as Maltese English, in recognition of the fact that it is influenced by the pronunciation, intonation, grammar and vocabulary of Maltese (Borg, 1988; Brincat, 2011; Krug and Sönning, 2018). On the other hand, spoken Maltese regularly features the use of English words, phrases, sentences and stretches of discourse (Borg, 1988; Camilleri Grima, 2013).

Monolingual input is highly unlikely for Maltese children (Vella, 2013). Stable bilingualism at a societal level and extensive language contact mean that children receive both Maltese and English input from a very early age, with amount and timing of exposure varying across households (Camilleri, 1995; Gatt and Dodd, 2019). Adults speaking Maltese to their young children often prefer English words or phrases over their Maltese equivalents (Borg, 1988). This ‘functional borrowing’ pattern characteristic of child-directed language use is potentially explained by the relatively simpler phonotactic structure of English, despite Maltese and English have similar consonantal phonetic inventories (Grech and Dodd, 2008; Galea and Ussishkin, 2018). This language choice pattern, specific to adult-child dyads, supplements the established borrowings, core borrowings and single-word code-switches from English expected in spoken Maltese (Gatt et al., 2011; see also Myers-Scotton, 2002, 2006). While established English borrowings compensate for lexical gaps in Maltese, e.g., *stiker* (sticker), core borrowings are English words employed predictably instead of available Maltese equivalents, as in the case of *toys* typically being preferred to *ġugarelli* in both adult- and child-directed contexts. Functional borrowing is thus a form of core borrowing specific to child-directed language use. On the other hand, single-word switches involve the sporadic preference of an English word to a Maltese equivalent. In this study, the participants’ Maltese-dominant home language exposure was established upon initial telephone contact with the primary caregiver and confirmed by the latter through completion of a language background questionnaire.

### 2.2. Language sampling

A naturalistic 20-min sample of children’s utterances was obtained as they engaged with their main caregivers in free play. A standard set of toys was provided to enhance replication of the sampling context across children. This consisted of a telephone, camera, abacus, stacking cups, cars, baby doll and baby care items, kitchen set, farm animals, tool set, insert puzzles and a pop-up cause-and-effect toy. The range of play materials was purposely chosen to cater for the varying levels of cognitive skill expected in the 12-30-month age range, besides taking children’s different toy preferences into account. Play interactions were audio- and video-recorded. Recordings were transcribed orthographically on the basis of the audio-recordings. Video-recordings were viewed when attempting to decipher unintelligible productions. For five (11%) of the language samples, all intelligible word tokens produced spontaneously and imitatively by the children were transcribed independently by a second transcriber, a qualified speech-language pathologist, who was provided with transcription guidelines and field notes taken during direct observation of the adult-child dyads. The mean percentage agreement between transcribed word tokens resulted in an inter-transcriber reliability value of 91.37%.

### 2.3. Caregiver report

Caregivers completed a Maltese-English adaptation of the vocabulary checklist of the first edition of the MacArthur Communicative Development Inventories: Words and Sentences (CDI: WS) (Fenson et al., 1993) for children exposed primarily to Maltese (Gatt, 2010). This consisted of 916 words, organized into 24 semantic categories. The inventory drew on the contents of the original U.S. English version (Fenson et al., 1993), Caselli and Casadio’s (1995) Italian adaptation, as well as actual words reported on earlier checklist versions for 12-30-month-old children raised in Maltese-speaking families. Maltese lexical items made up 68.45% of the checklist entries while English words comprised 27.29%. The rest (4.26%) were words that were not clearly identifiable as Maltese or English, such as sound effects, homophones and cognate terms, hence referred to as ‘non-specific language words’. The lexical items in semantic categories covering content words were presented in Maltese and/or English according to reported usage during piloting of the checklist adaptation. Here, English words were consistently fewer than Maltese words, with discrepancies varying in size depending on the semantic category. For example, the Animals section contained 28 Maltese words and 22 English words, whereas the Action words category listed 78 words, of which 70 were Maltese (see Gatt et al., 2011). In the function word categories, Maltese and English translation equivalents were available for most semantic concepts. Among the checklist entries, 215 pairs of Maltese and English words corresponded to the same meaning in adult language use. Each semantic category included a recall section in which caregivers could add words in their children’s expressive repertoires not listed in the checklist. Gatt et al. (2014) reported vocabulary checklist scores to correlate positively and significantly with the number of word types produced spontaneously during play (*r* = 0.635, *p* < 0.001) and with the number of picture labels elicited through an informal naming task (*r* = 0.556, *p* < 0.001), providing evidence for the checklist adaptation’s concurrent validity.

Primary caregivers also completed a language background questionnaire developed purposely for the study. Although the psychometric properties of the questionnaire were not evaluated, the instrument served to document each child’s language exposure and confirm the predominant use of Maltese in the home, as claimed by children’s caregivers at the recruitment stage. Questions addressed caregivers’ Maltese and English proficiency and use, children’s relative exposure to Maltese and English on a daily basis and language mixing patterns used with and around each child. Bilingual oral language and literacy skills were reported by 95.5% (*N* = 42) of the caregivers, with the remaining two reporting limited proficiency in English. Just over half of the respondents (52.3%, *N* = 23) reported speaking Maltese more confidently than English, with the rest feeling equally comfortable speaking both languages. Varying degrees of language mixing were reported in the children’s language exposure, confirming the likelihood that none of the participants were exposed to monolingual Maltese and monolingual English input. Informal observation of adult language use patterns during play confirmed lexical mixing to be employed by all caregivers, including the two mothers having limited bilingual proficiency. All respondents reported that their children were addressed in Maltese for over 60% of the time on a daily basis.

### 2.4. Procedure

Data were collected in the children’s homes over two sessions, one to two weeks apart. During the first session, details of the child’s birth history, general health, physical and language development, parental education and occupation, as well as any family history of language impairment, were obtained during an interview with the main caregiver. The questionnaire and vocabulary checklist were then discussed and their completion solicited by the next visit, which took place one to two weeks later. In order to enhance accuracy in vocabulary reporting, each caregiver was briefed about the purpose of the checklist and taken through the bilingual guidelines attached to the tool. It was emphasized that only words used spontaneously by children were to be reported. During the second visit, each child’s expressive language was sampled during 20 minutes of free play with the caregiver.

Preliminary transcription of children’s vocalizations during free play was attempted as the caregiver-child dyads were observed. A full orthographic transcription of spontaneous and imitated utterances was subsequently carried out on the basis of the audio recordings. Video recordings helped decipher unintelligible productions captured on the audio recordings. The present study focused only on the spontaneous utterances, which amounted to 86% of all transcribed productions. Excerpts of nursery rhymes and songs were not transcribed.

### 2.5. Data coding

In this study, our primary focus was on words having a commonality of 50%+, that is, words produced by at least half the children in the whole cohort and in each age group. These ‘more commonly produced words’, as documented separately by checklist and sample data, were identified in relation to the age point/s at which they were produced. In each age group, the more common words were classified according to a commonality score ranging between 6 and 11. The score of 5 was only relevant to the 30-month-olds, since it represented 50% of the 10 children in this group. Since the other age groups were slightly larger, counting 12 (18-month-olds) and 11, their lowest commonality score was 6. Similarly, the highest score of 12 was only relevant to the 18-month-olds, with 11 being the maximum score for the 12-, 18-and 24-month-olds and 10 for the 30-month group. Words not reaching the designated commonality threshold were coded for the number of occurrences, but were not analyzed further.

#### 2.5.1. Caregiver report measures

Reported words were entered as variables in an item-by-child database. Words ticked on the checklist, as well as recalled words, the additional items contributed by some caregivers in dedicated boxes attached to each semantic category, were all considered as variables. Recalled words were tagged accordingly, to distinguish them from recognized words. Including recalled words in checklist measures enhanced comparability with language sample data as it compensated somewhat for the predetermined number of word recognition opportunities provided by the checklist. All reported words were tallied individually and a *commonality score* was derived for each item. This represented usage across the cohort, in terms of the number of children reported to produce each word. For every child, a *Total Vocabulary (TV) score*, which summed all recognized and recalled words reported on the checklist, was computed. This represented the child’s vocabulary size as indexed by the caregiver.

#### 2.5.2. Sampled words

Spontaneous vocalizations conveying consistent meanings were identified as words. These comprised forms which approximated adult targets closely and others which showed reduced phonological complexity but still matched at least two consecutive phonemes of the adult target (see Huttenlocher et al., 1991). Meaningful use was established on the basis of children’s preceding and subsequent utterances, focus of attention and accompanying gestures. Stability in meaning was determined if a sound-meaning pairing occurred more than once in the same sample and/or was recognized by the caregiver. Productions bearing no resemblance to an adult form, despite consistency in meaning, were assigned word status but were not analyzed in this study, in view of the decreased likelihood of them having counterparts in the checklist data. Productions were classified as unintelligible if three consecutive attempts at deciphering them were unsuccessful. Meaningful interjections (e.g., *ohoh*), sound effects (e.g., *brmmbrmm*) and routine words (e.g., *baħħ* [all gone]) were coded, but fillers (e.g., *emm*) and part-word repetitions were not. Incomplete lexical items were only inputted if the target word was unequivocally obvious. Instances of jargon were not coded. A Number of Different Words (NDW) score, tallying types (the sample-based counterpart to the TV score calculated for the checklist data), and the Total Number of Words (TNW, token count) were computed for every child. In the case of samples manifesting emergent grammar, word components having lexical-semantic meaning were counted as lexical items in their own right. Thus, Maltese enclitic pronouns attached to nouns, verbs and prepositions were coded as separate lexical items (e.g., *xagħri* [*xagħr* + *−i*] (hair + my) = 2 types), even when inflecting for number and/or gender, e.g., with *xagħru* [*xagħr* + −*u*] (hair + his) and *xagħrha* [*xagħr* + −*ha*] (hair + her), both instances were counted as two words, even if they occurred in the same sample. In contrast, word elements having solely grammatical meaning, such as gender and number inflections on verbs and adjectives, were tallied as tokens of the same type. In this vein, demonstrative pronouns inflecting for number and gender were not coded as unique words, e.g., *dan, din* (this m./f.) and *dawn* (these) were coded as *dan/din/dawn* (1 type, 3 tokens). Number words such as ‘eight’, ‘five’ and so on were coded collectively as one type, ‘one, two, three…’. Word combinations employed invariably as a single lexical item to convey a specific meaning, e.g., *love you,* were coded as one word if the two components did not also appear individually in the same sample. Maltese enclitic pronouns suffixed to the same noun or verb and not appearing elsewhere in the sample, as in the case of the indirect object pronoun *-lu* (to him) and *biddel* (change) in *biddillu* (change (to) him), were also coded as one word. Some children embedded English words in Maltese grammatical constructions, in which case coding conventions described here were applied as necessary, e.g., *ball dak… dik pupa* (that (m.) (is) a ball… that (f.) (is) a doll) was coded as *ball*, *dak/dik*, *pupa* (NDW = 3; TNW = 4).

Sampled words that matched any of the 916 words on the vocabulary checklist were tagged according to the semantic categories on the latter. For example, identification of ‘car’ in a sample led to it being tagged as ‘Vehicles – Real or Toy’. Sampled words that were not available for reporting on the checklist were assigned to one of the 24 semantic categories deemed to be most suited to the word’s semantic function and tagged as <Specific category name>_Other’. Thus, spontaneous production of ‘bicycle’, a lexical item not on the vocabulary checklist, was identified as ‘Vehicles – Real or Toy_Other’. For each child, identified types were assigned a score of 1 every time they were produced, enabling computation of the *frequency of occurrence* in the specific age group and across the composite sample. The *commonality score* of each word represented the number of children using it at least once.

#### 2.5.3. Coding for grammatical categories

All inputted words were coded as either social words (sound effects, routine words), nouns, verbs, adjectives or function words, the latter including adverbs, pronouns, question words, prepositions, articles and quantifiers, negative markers and conjunctions. The auxiliary verbs *qed, qiegħed*, and *qiegħda*, together with the future particles *ħa* and *se*, were coded as function words. The relevant analytical framework was adopted from Caselli et al.’s (1999) study of grammatical categories in English and Italian and, accordingly, considered as a simplified rendition of the linguistic input received by children.

#### 2.5.4. Coding for language

All items were also coded as either Maltese, English or non-specific language words, drawing on the language contact phenomena expected in Maltese children’s input (see Section 2.1). Items tagged as Maltese were native Maltese words, as well as established English borrowings that had no Maltese equivalent, e.g., *kompjuter* (computer), ‘hello’. The remaining English words were coded as English items since they were preferred to the Maltese equivalent. Examples included ‘colours’ instead of *kuluri* and ‘thank you’ for *grazzi*. Examples of non-specific language words included banana, blu and basket.

## 3. Results

Aggregate vocabulary scores derived from checklist and sample data were examined in a preliminary analysis. Word-level data from each source were then examined separately, followed by comparative analyses. Throughout, the focus was on the words employed by 50% or more of the participants.

Table 1 shows descriptive information for Total Vocabulary (TV), Number of Different Words (NDW) and Total Number of Words (TNW) scores, each of which measured the full range of words produced, regardless of commonality. As expected, all sum and mean scores increased with age. TV values were consistently larger than sampled vocabulary scores, with Wilcoxon signed-rank tests showing mean differences to be significant (TV and NDW: *Z* = −5.78, *p* < 0.001, TV and TNW: *Z* = −3.93, *p* = < 0.001). On average, the TNW score, measuring all spontaneous occurrences of sampled words, was also significantly larger than NDW, which tallied the unique words (*Z* = −5.30, *p* < 0.001). Yet, partial correlations between TV and NDW, the aggregate scores quantifying reported and sampled vocabulary size respectively, yielded moderately positive and significant relationships (*r* = 0.626, *p* < 0.001) when age was partialled out. A close correspondence between checklist and sample measures was therefore present, with children’s scores ranking similarly across both methods despite the numerical differences. The coefficient between TV and TNW counts was also significant but lower than for NDW (*r* = 0.454, *p* < 0.05). This could be because TNW scores were not a direct counterpart to TV since they incorporated all occurrences of words produced. These introductory results set the scene for the subsequent item-based analyses, establishing that the starting point for these was a statistical correspondence between vocabulary size as gauged by both methods.

**Table 1:** Mean, standard deviation (SD), and sum of scores for Total Vocabulary (TV), Number of Different Words (NDW), and Total Number of Words (TNW) in relation to age.

Age (months)	*N*	TV			NDW			TNW		
		Sum	Mean	SD	Sum	Mean	SD	Sum	Mean	SD
12	11	229.00	20.82	23.66	31	2.82	4.29	89	8.09	13.10
18	12	816.00	68.00	73.52	163	13.58	11.22	585	48.75	46.19
24	11	2473.00	224.82	161.74	315	28.64	22.65	1,038	94.36	79.42
30	10	4330.00	433.00	163.87	943	94.30	23.85	3,359	335.90	110.39

### 3.1. Word usage as reported by caregivers

Table 2 lists the 43 words produced by at least 50% of the 44 participants, according to their caregivers. Commonality, expressed in terms of absolute and proportion scores, had an upper ceiling of 43 (97.7%). Words were spread across 12 semantic categories, with the most commonly employed being Daily experiences (*N* = 15), People (*N* = 8) and Sounds (*N* = 6). The five words reported for at least 75% of the cohort represented People and Daily experiences. The words mamà/mummy, papà/daddy and *nanna* (grandma), all belonging to the People category, were the most commonly used overall. Additional words recalled by caregivers were generally reported for only small numbers of children, explaining why none appeared in the list of most commonly reported words for all the participants. The most common recalled items were ‘cereal’ and ‘medicine’, each produced by 11.4% (*N* = 5) of the sample, while ‘bread’, ‘eyes’, ‘good night’, ‘sorry’ and *tersaq* (move) were reported for four children (9.1%). A total of 64 words, spanning 10 semantic categories, were never reported. Of these, 14 (21.9%) were Maltese and 50 (78.1%) were English, with three words in each language being equivalents. All the English items were function words. The latter totaled 60 (93.8%) of the unreported words and were accompanied by three nouns and one adjective.

**Table 2:** The more commonly reported words across participants (*N* = 44, age range = 12–30 months) with respective checklist semantic category and raw (%) commonality score.

Reported word	Semantic category	Commonality (%)	Reported word	Semantic category	Commonality (%)
mamà, mummy	People	43 (97.7)	shoes	Clothing	25 (56.8)
papà, daddy	People	41 (93.2)	amm amm	Sounds	25 (56.8)
*nanna* (grandma)	People	39 (88.6)	*taqa’* (fall)	Action words	24 (54.5)
bye, ciao, tatà	Daily experiences	35 (79.5)	*qalbi* (‘my heart’)	Daily experiences	24 (54.5)
*aħħ* (ouch)	Daily experiences	33 (75.0)	bird	Animals	24 (54.5)
one, two, three…	Daily experiences	32 (72.7)	fish	Animals	24 (54.5)
*bumm* < loud sound>	Sounds	31 (70.5)	*mimmi* (‘pain’)	Daily experiences	24 (54.5)
thank you	Daily experiences	30 (68.2)	*baħħ* (all gone)	Daily experiences	24 (54.5)
book	Everyday objects	30 (68.2)	boy	People	23 (52.3)
ball	Toys and games	30 (68.2)	please	Daily experiences	23 (52.3)
wuw wuw < dog sound>	Sounds	30 (68.2)	*le* (no)	Daily experiences	23 (52.3)
no	Daily experiences	29 (65.9)	muu < cow sound>	Sounds	23 (52.3)
kokò	Daily experiences	28 (63.6)	mmm	Sounds	23 (52.3)
*nannu* (grandpa)	People	28 (63.6)	*bumma*	Daily experiences	23 (52.3)
*(piff) jaqq* < expression indicating disgust>	Daily experiences	28 (63.6)	*xita* (rain)	Outside	22 (50.0)
pipì	Daily experiences	27 (61.4)	fish	Food and drink	22 (50.0)
car	Vehicles	27 (61.4)	banana	Food and drink	22 (50.0)
miao	Sounds	27 (61.4)	child’s name	People	22 (50.0)
dog	Animals	26 (59.1)	*bravu, brava* (good m., f.)	Descriptive words	22 (50.0)
baby	People	26 (59.1)	*dudu* (worm)	Animals	22 (50.0)
hello	Daily experiences	26 (59.1)	kiss	Action words	22 (50.0)
pet’s name	People	26 (59.1)			

Commonality threshold = 22 (50.0%). Italics denote *Maltese* words, small capitals denote non-specific language words.

In the analysis by age group, words that reached a commonality of 50% and over totaled 617. Supplementary Table S1A lists the words more commonly reported by caregivers when a lower commonality threshold of 50% was applied. Word usage increased with age, so that older children added more items to their frequently-used repertoire. As expected, more words were present at the lower end of the commonality range (50%+), with numbers tapering off at higher commonalities. Relatively few words were produced by all children in each age group.

**Figure 1:**
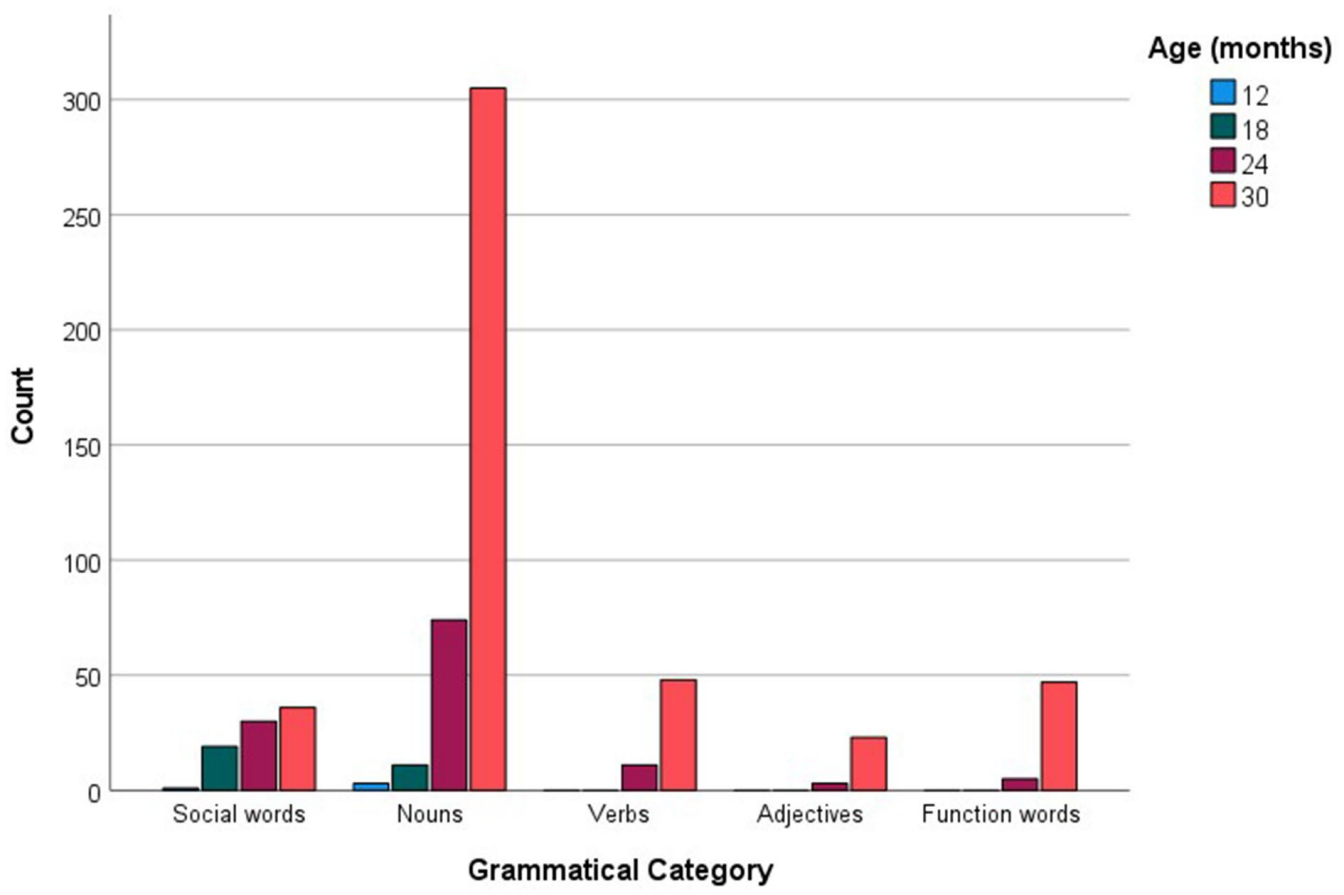
Grammatical category counts (Social words, Nouns, Verbs, Adjectives, Function words) as a function of more commonly reported words at 12, 18, 24, and 30 months.

**Figure 2:**
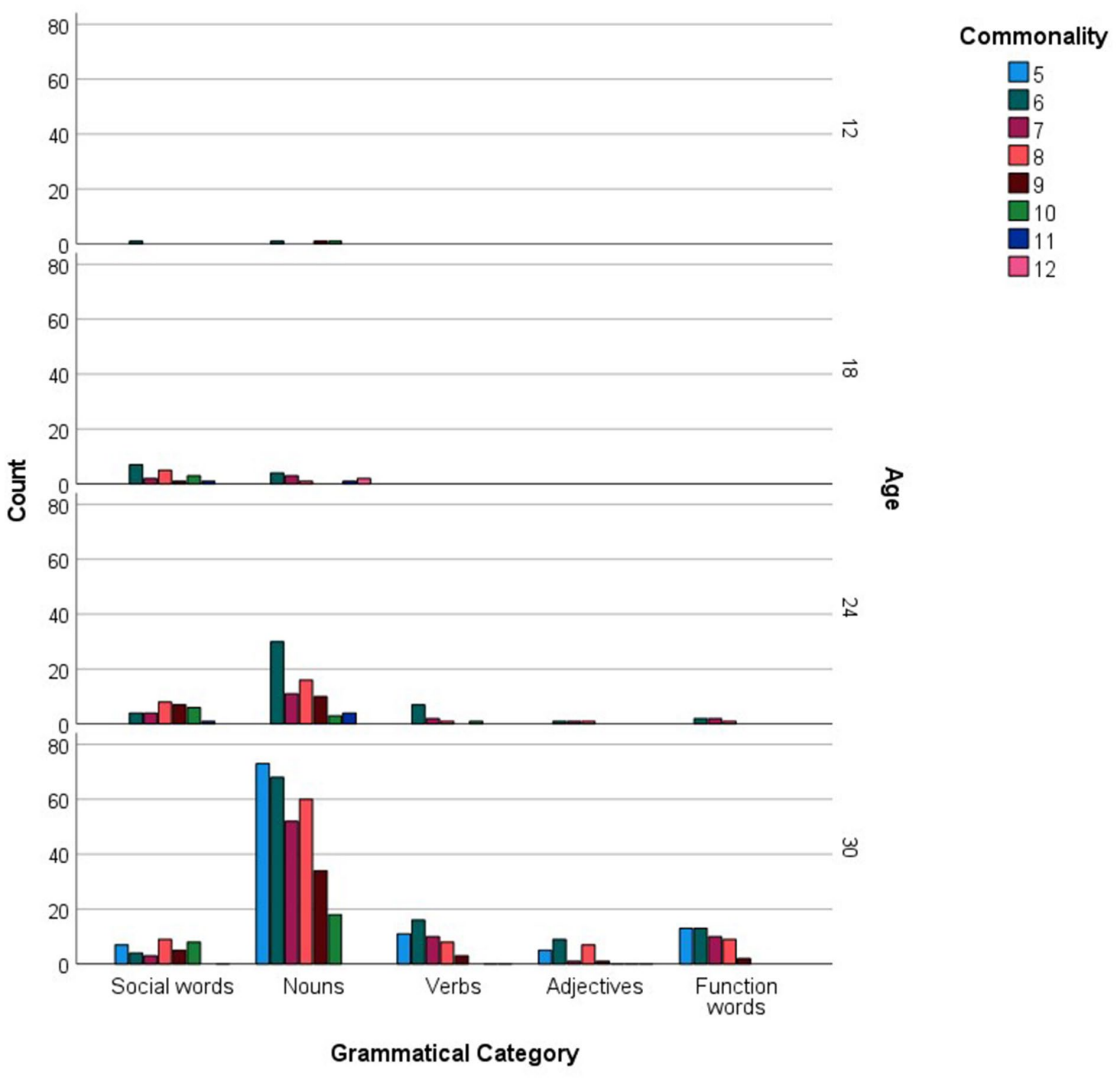
Grammatical category counts (Social words, Nouns, Verbs, Adjectives, Function words) as a function of more commonly reported words, including commonality levels (i.e., numbers of children using each word), at 12, 18, 24, and 30 months.

**Figure 3:**
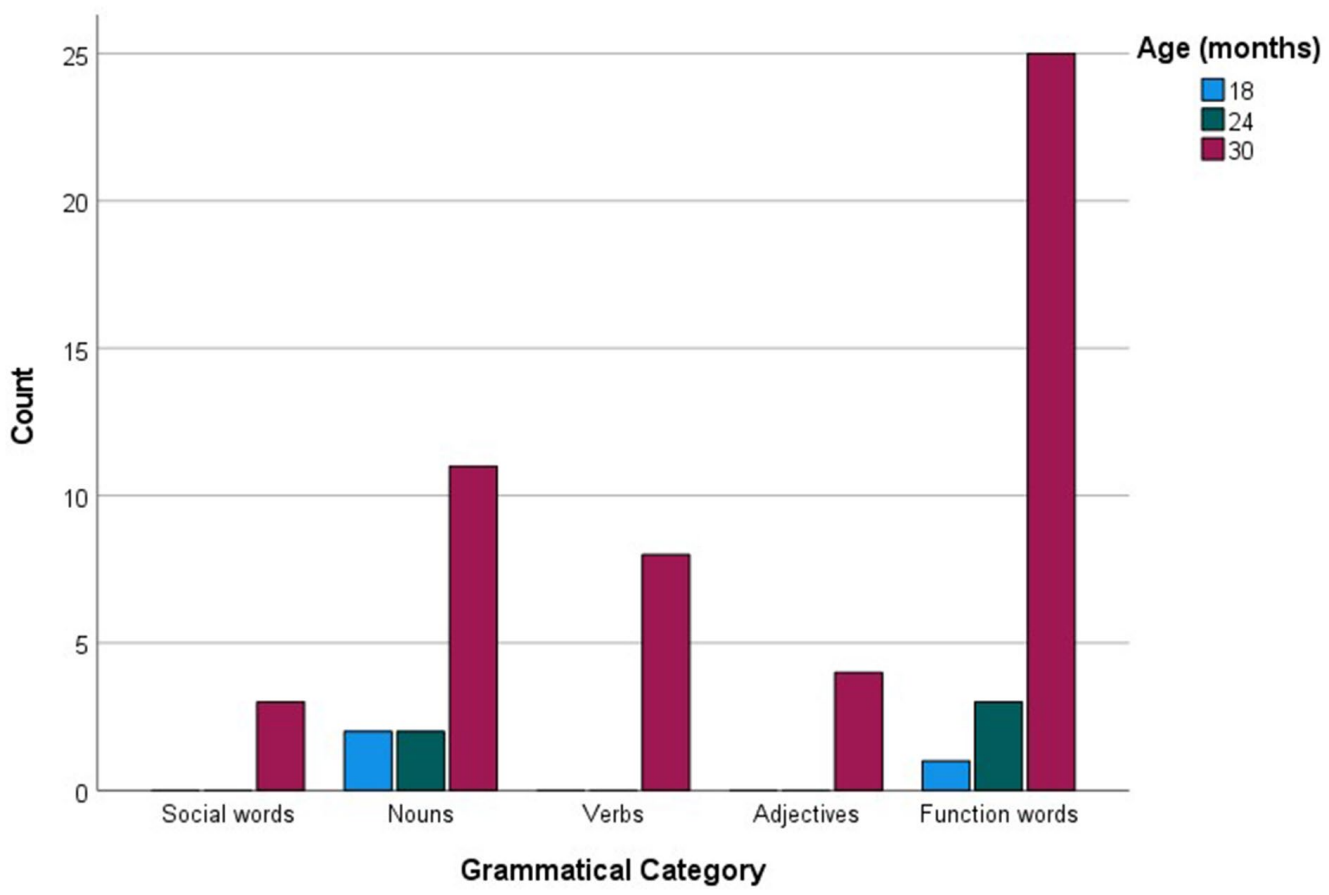
Grammatical category counts (Social words, Nouns, Verbs, Adjectives, Function words) as a function of more commonly sampled words at 12, 18, 24, and 30 months.

**Figure 4:**
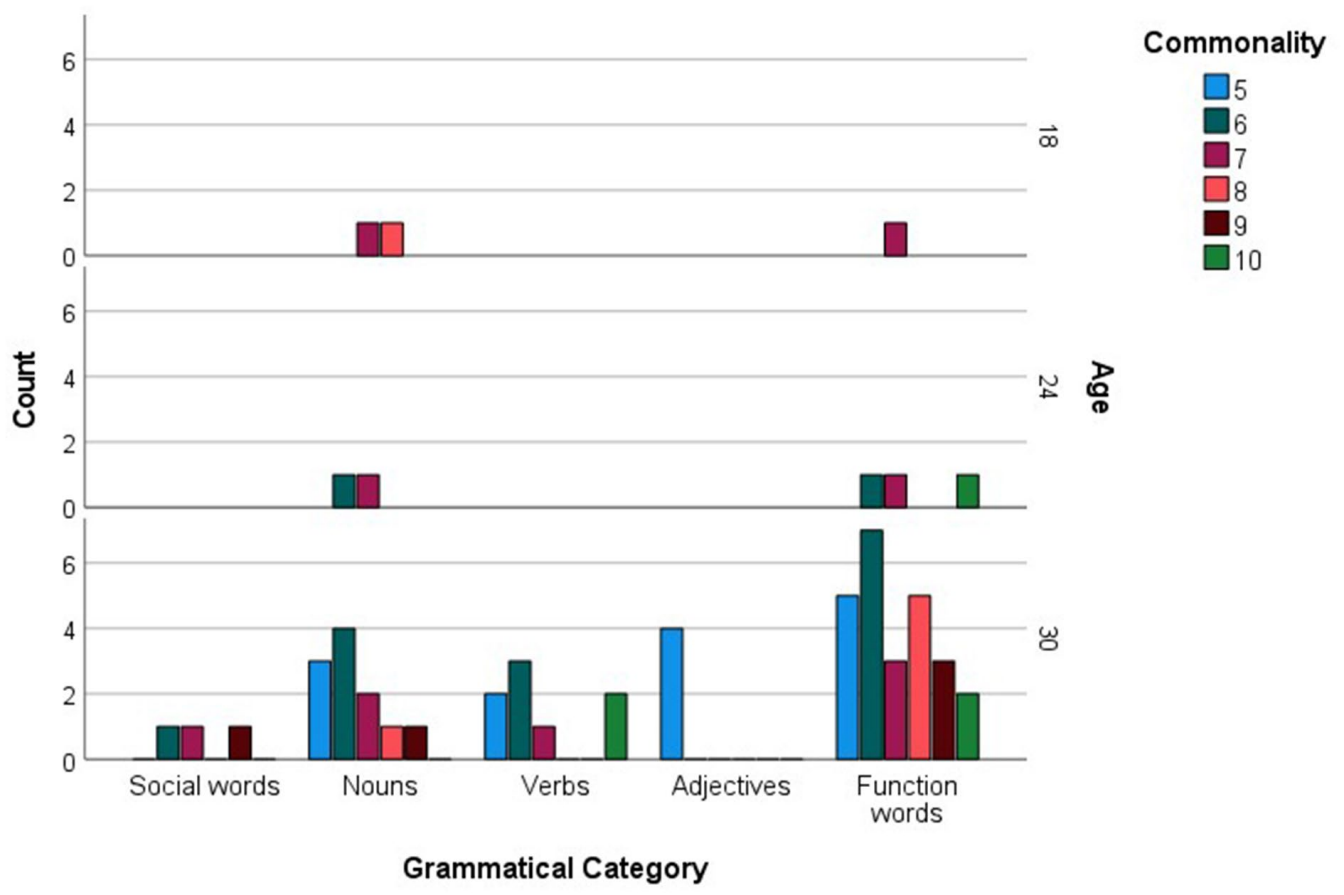
Grammatical category counts (Social words, Nouns, Verbs, Adjectives, Function words) as a function of more commonly sampled words, including commonality levels (i.e., numbers of children using each word), at 12, 18, 24, and 30 months.

For every age group, the more commonly reported words were analyzed in relation to the grammatical categories and languages they represented. Figure 1 shows the distribution of social words, nouns, verbs, adjectives and function words across the more commonly reported checklist words, while Figure 2 inspects the grammatical category trends more closely, zooming in on the different levels of commonality embraced within every category for each age point. Across all commonality levels, social words increased considerably between 12 and 18 months but only increased marginally at subsequent age points. Nouns increased exponentially throughout each phase between 12 and 30 months. In contrast, verbs, adjectives and function words were absent at 12 and 18 months but increased exponentially from 24 to 30 months. The distribution of languages across the more commonly reported words for each age point is shown in Figure 3, with Figure 4 showing the Maltese, English and non-specific language words for each commonality level. Commonalities of Maltese and English words were comparable at 18 and 24 months. At 30 months, the number of Maltese words among those most commonly reported increased drastically.

### 3.2. Sampled word usage

Our next analysis addressed the commonality and frequency of words sampled during free play. At 12 months, no words reached the commonality threshold of 50%+, reflecting the limited production of words among the youngest participants. Words produced by >50% of the participant group (*N* = 22) were limited to two (see Table 3). The words *dan, din* [this (m., f.)] and *dawn* (these), coded as a single type, were produced by 63.6% (*N* = 28) of the participants. This item also placed highest in terms of frequency of production across all word tokens by all children (8.5%), which is understandable given that, in effect, the frequencies of three tokens were collapsed into a single frequency score. Mamà, mummy appeared at least once in the language samples of 21 children, ranking in fifth place overall in terms of frequency (2.4%). Other frequently produced words were *hawn, hawnhekk* (here) (3.8%), *il-, l-* and other definite articles (3.2%), together with *iva, eħe* (yes) (2.6%). Table 4 lists the 10 most frequently occurring words across all samples, in order of descending frequency. Generally, the more frequently produced words were also the more common, although only *dan, din, dawn* surpassed our commonality threshold. The word *fejn* (where) appeared to be an outlier, ranking 7th in composite frequency with a commonality of 8.

**Table 3:** The more commonly sampled words across participants (*N* = 44, age range = 12–30 months) with assigned semantic category, raw (%) commonality score and composite frequency, expressed as a proportion of the composite Total Number of Words (i.e., 5,071) across all age groups.

Sampled word	Semantic category	Commonality (%)	% Composite frequency
*dan, din, dawn* (this (m., f.), these)	Pronouns	28 (63.6)	8.5
mamà, mummy	People	21 (47.7)	2.4

Commonality threshold = 22 (50.0%). Italics denote *Maltese* words, small capitals denote non-specific language words.

**Table 4:** The more frequently sampled words across participants (*N* = 44, age range = 12–30 months) with assigned semantic category, composite frequency, expressed as a proportion of the composite Total Number of Words across all age groups (i.e., 5,071), and raw (%) commonality score.

Sampled word	Semantic category	% Composite frequency	Commonality (%)
*dan, din, dawn* (this (m., f.), these)	Pronouns	8.5	28 (63.6)
*hawn, hawnhekk* (here)	Prepositions and locations	3.8	20 (45.5)
*il-, l-…* (def. Art.)	Quantifiers	3.2	11 (25.0)
*iva, eħe* (yes)	Daily experiences	2.6	16 (36.4)
mamà, mummy	People	2.4	21 (47.7)
*dak, dik, dawk* (that (m., f.), those)	Pronouns	1.9	17 (38.6)
*fejn* (where)	Question words	1.8	8 (18.2)
one, two, three…	Daily experiences	1.8	14 (31.8)
*tara* (see)	Action words	1.8	15 (34.1)
hello	Daily experiences	1.7	13 (29.5)

Italics denote *Maltese* words, small capitals denote non-specific language words.

**Figure 5:**
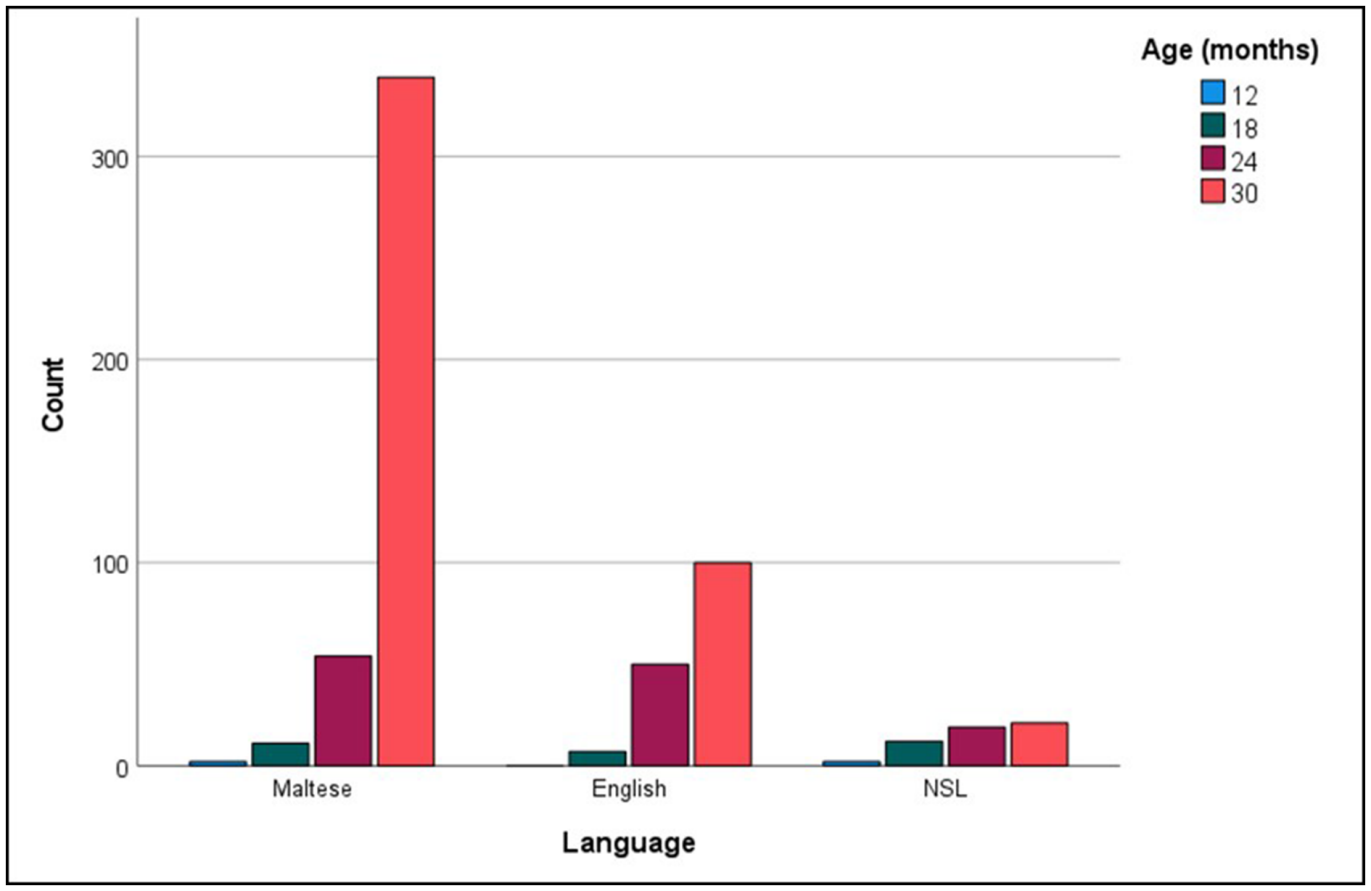
Language counts (Maltese, English and non-specific language (NSL) words) as a function of more commonly reported words at 12, 18, 24, and 30 months.

**Figure 6:**
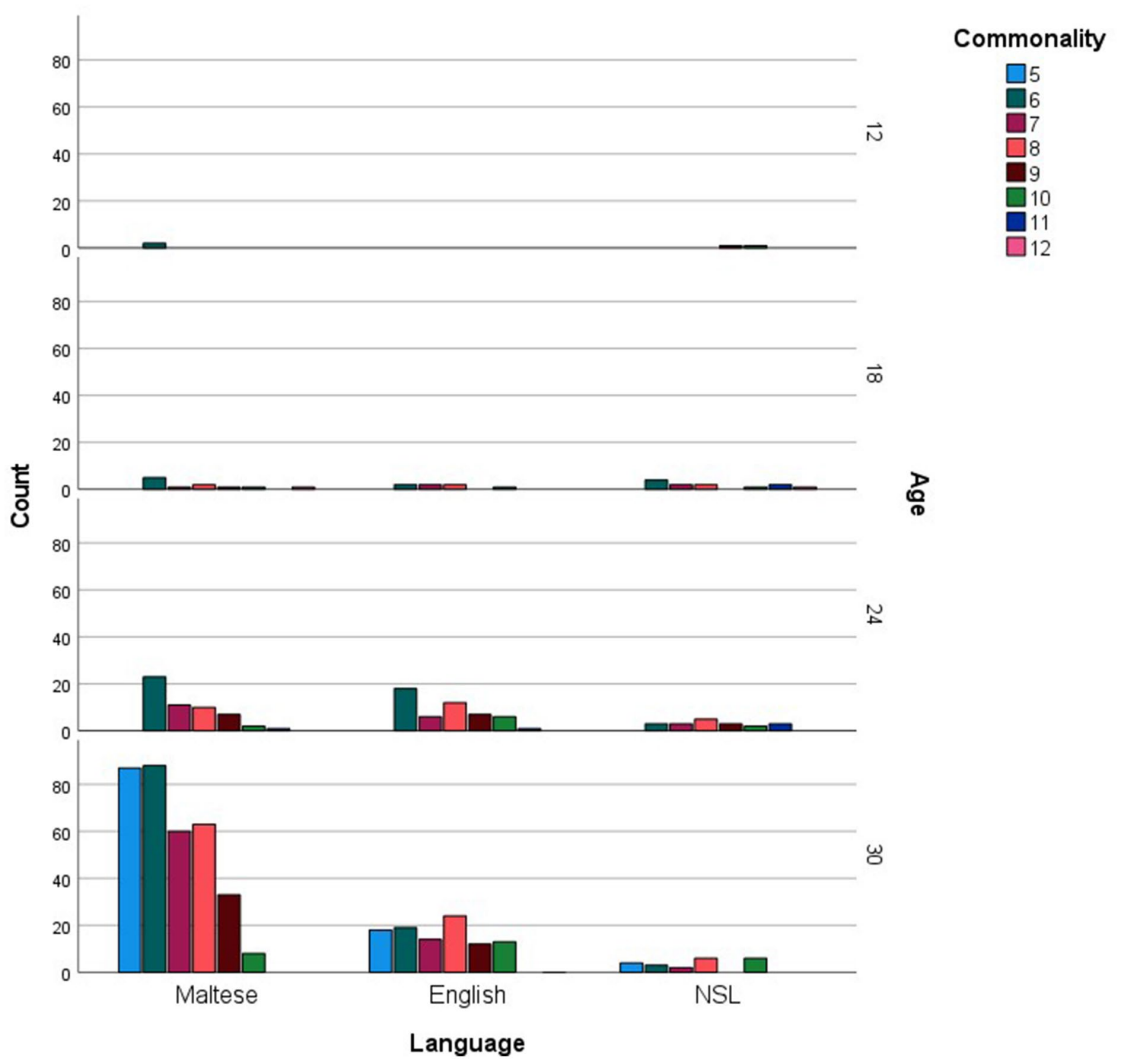
Language counts (Maltese, English and non-specific language (NSL) words) as a function of more commonly reported words, including commonality levels (i.e., numbers of children using each word), at 12, 18, 24, and 30 months.

**Figure 7:**
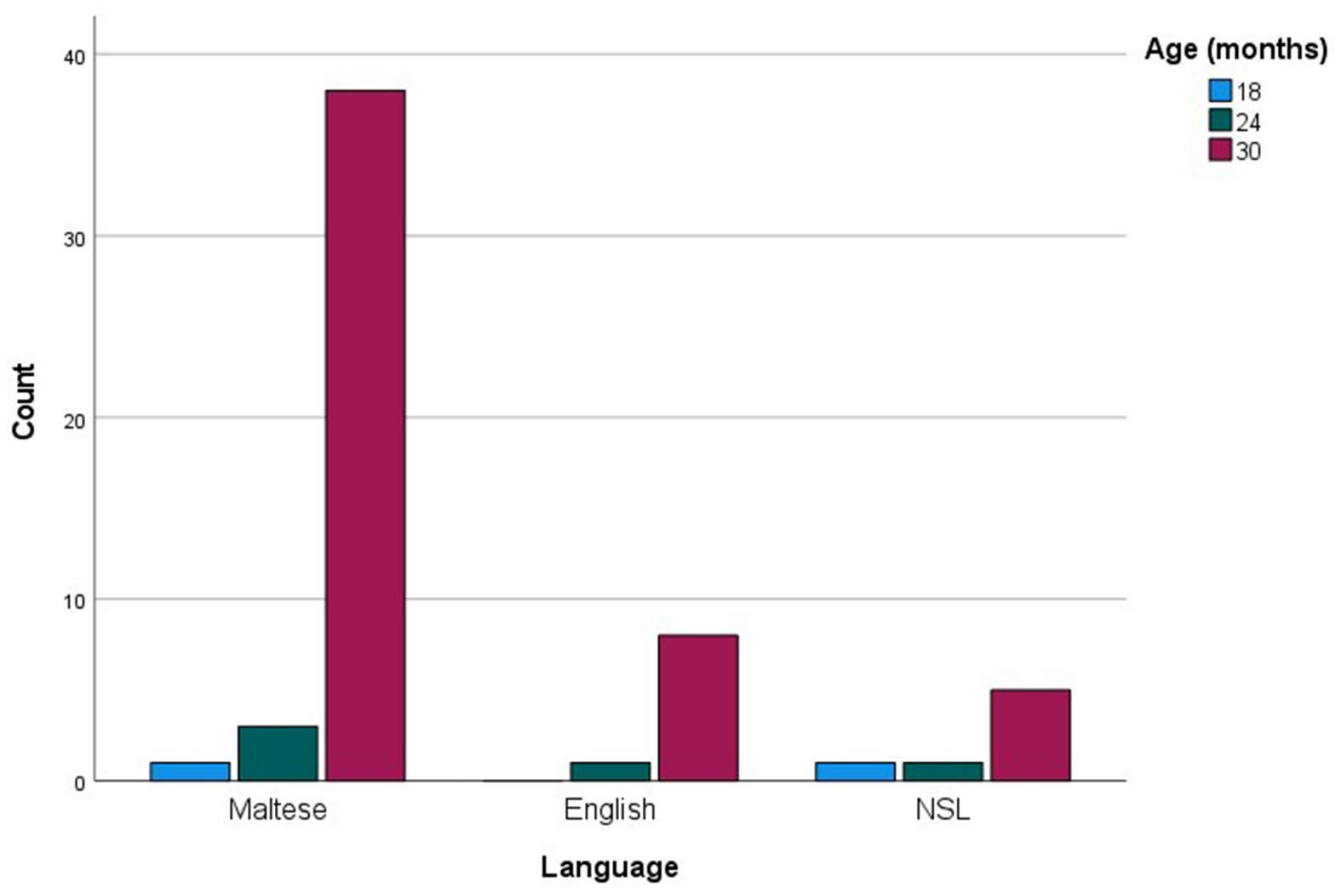
Language counts (Maltese, English and non-specific language (NSL) words) as a function of more commonly sampled words at 12, 18, 24, and 30 months.

**Figure 8:**
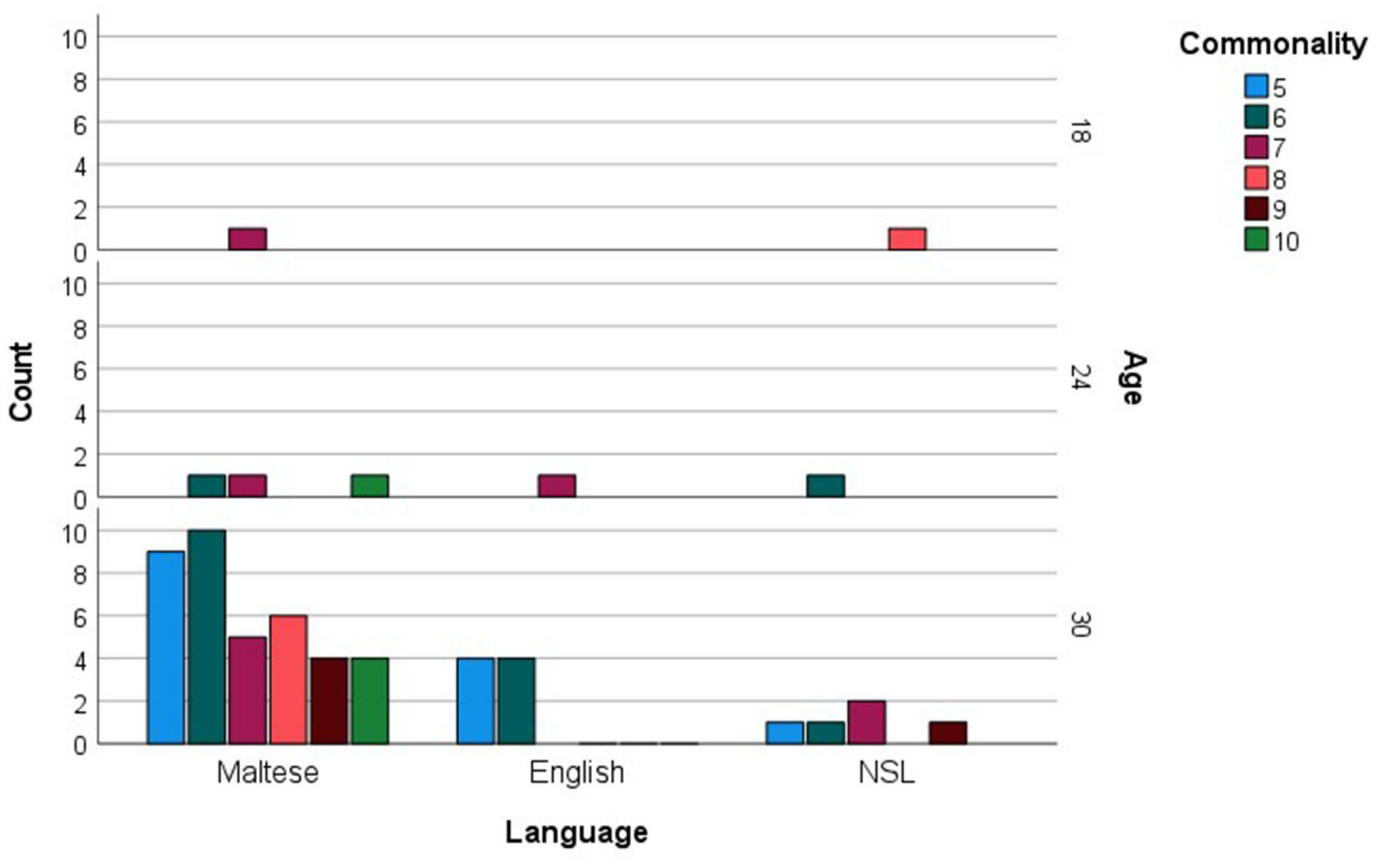
Language counts (Maltese, English and non-specific language (NSL) words) as a function of more commonly sampled words, including commonality levels (i.e., numbers of children using each word), at 12, 18, 24, and 30 months.

When analyzed in relation to age, commonly produced words were much fewer in number compared to checklist data, amounting to 58 (see Supplementary Table S1B). For each age group, the more common words were then examined as a function of their grammatical categories (see Figure 5). Social words, verbs and adjectives were among the more commonly sampled words only at 30 months, while nouns were among the more commonly produced words at 18 and 24 months, reaching maximum commonality at 30 months. The most prominent trend, however, is a sheer increase in commonly produced function words at 30 months. Figure 6 presents the breakdown of commonality scores constituting the trends illustrated in Figure 5. When analyzed in relation to language, the more commonly sampled words were somewhat balanced across Maltese and English for the 18-and 24-month-olds (see Figures 7, 8). At 30 months, Maltese words were clearly predominant, while the relative increment in English words was more protracted.

### 3.3. Comparison of item-level results for checklist and sample data

Our final analysis compared main trends emerging in the checklist and sample datasets. Of the more commonly sampled words, 28 items had a matching counterpart in the checklist dataset. These items, together with age and commonality of occurrence, are listed in Table 5. While the number of item-level matches is extremely limited, particularly in comparison to the expansive checklist figures, correspondences are remarkably close for 30-month words, even in terms of commonality score. In terms of grammatical composition of the more commonly produced words, Figures 1, 3 revealed elements of a similar shift in emphasis across methods, although the captured stages are different. Notably, the sample data portray the full wave of reorganization from social words to nouns, verbs and adjectives and subsequently function words in the 30-month profiles (see Figure 3). In the checklist data, on the other hand, 24-and 30-month more commonly produced words seem to be at the point of shifting to a predominance of function words (Figure 1). When partialling out the effects of age, a significant albeit low correlation resulted (*r* = 0.293, *p* < 0.001), indicating similar trends overall, despite the differences in numerical values. In comparison, caregiver report and language sampling were more compliant in profiling the distribution of languages among the more commonly produced words. Both methods identified comparable numbers of English and Maltese words among the lexical items more commonly produced by 18-and 24-month-olds, as well as a predominance of Maltese words at 30 months (Figures 5, 7). A partial correlational analysis that controlled for age effects revealed a remarkably high and significant coefficient (*r* = 0.935, *p* < 0.001).

**Table 5:** Matching words across checklist and sample datasets, with age of each occurrence and commonality score for each.

Word	Sample occurrence (age, commonality)	Checklist occurrence (age, commonality)
*dan, din, dawn* (this (m., f.), these)	24, 10 30, 10 18, 7	24, 8 30, 8
*hawn, hawnhekk* (here)	30, 10 24, 7	30, 7 24, 7
*taqa’* (fall)	30, 10	24, 10
*tara* (see)	30, 10	30, 5
*iva, eħe* (yes)	30, 9	24, 7 30, 8
*dak, dik, dawk* (that (m., f.), those)	30, 9 24, 6	30, 10
*telefon*	30, 8	30, 9
*ħa, se* (fut. particle)	30, 8	30, 6
*jiena* (I)	30, 8	30, 10
*hekk* (so)	30, 8	24, 5
mamà, mummy	18, 8 30, 7	12, 11 18, 12 24, 11 30, 10
*le* (no)	30, 7	24, 9 30, 9
papà, daddy	30, 7	12, 9 18, 11 24, 11 30, 10
*tiġi* (come)	30, 6	30, 9
*one, two…*	30, 6	18, 10 24, 10
car	30, 6	24, 11 30, 9
toy	30, 6	24, 9 30, 8
*fejn* (where)	30, 6	30, 10
*iċċempel*	30, 6	30, 7
elephant	30, 6	30, 7
*x’, xiex* (what)	30, 6	30, 5
*tajjeb, tajba* (good)	30, 5	30, 8
*għax* (because)_	30, 5	30, 6
*mela* (so)	30, 5	30, 5
green	30, 5	30, 5
*għandek* (have)	30, 5	30, 6
blu	30, 5	30, 6
te’	30, 5	30, 9

Italics denote *Maltese* words, small capitals denote non-specific language words.

## 4. Discussion

The current study set out to identify word usage trends in a cohort of 12-30-month-olds who were predominantly exposed to Maltese in their homes, within a broader context of societal bilingualism. Its purpose was to compare word-level data identified through caregiver report and language sampling employed with the same children, in order to derive methodological implications that could guide theoretical understanding, as well as reporting instrument revision. To our knowledge, the comparison of word usage as determined by different methods for the same children is as yet unprecedented. The present investigation therefore attempted to add fresh insight to the long-debated issue of methodological bias in the measurement of young children’s early vocabularies. While word-level analyses have long contributed to and complemented the substantial research base relating to vocabulary acquisition, their relevance might at times be overlooked because of the scrutiny of individual items required for this purpose. Moreover, a direct comparison of individual items sampled directly and reported by parents contributes important theoretical and methodological insights. Specifically, by focusing on more commonly produced words in Maltese children, this study not only documented trends and patterns in word usage in an under-researched language pair, but also attempted to add depth to current views on the effectiveness of the parental report method.

The first set of preliminary findings showed significant differences between mean scores tallying reported and sampled vocabulary size, but also positive and significant correlations between them. Total Vocabulary (TV) and Number of Different Words (NDW) scores both addressed the range of words used, albeit through different sources. Thus, it was relatively unsurprising that the more commonly reported and sampled words, different in nature from TV and NDW scores but directly related, correlated positively and significantly too, despite their different numerical bases. While the nature of the data collection method inevitably impacts the numbers and range of words picked up, it is encouraging that, in terms of more common words across participants, similarities were documented. The correlational analyses in particular indicate that in general, the caregivers were able to report on usage of grammatical categories and languages in ways that ranked similarly to sampled trends.

The clear predominance of nouns in the checklist profiles reported here was somewhat predicted. Published core vocabulary lists have been compared to the CDI vocabulary checklist words, the latter taken as a representation of the words young typically-developing language learners are expected to use. Parallel studies by Frick Semmler et al. (2023) and Laubscher and Light (2020) both flagged a mismatch between the contents of core word lists and vocabulary checklists. The relative predominance of function words and scarcity of content words typical of core word lists conflicted with the distribution of grammatical categories expected in early vocabularies. The results we obtained for commonly reported words also suggest a noun bias, although this may be more related to caregiver reporting style than to the reporting tool itself. This is hypothesized on the basis of the bilingual vocabulary checklist employed in this study. The instrument employed had quantitatively similar noun and function word proportions since the function word categories generally presented items in both languages, effectively doubling the number of reporting opportunities, whereas noun categories contained much fewer equivalent terms. Therefore, while a noun bias is clearly evident in the reported data, a component of it could have stemmed directly from the caregivers’ filtering of reported information (Stiles, 1994). The contrasting predominance of function words manifested in the common words sampled adds further weight to this possibility.

The predominance of Maltese words in both checklist and sample datasets is perhaps one of the more interesting findings of this study. When transposed onto the grammatical category analysis, the inclination to produce more commonly occurring words in Maltese indicates that this is the language in which most of the nouns favored in the checklist dataset and the function words dominating the sample scores were produced. This implies that, for these children, Maltese was the more consistently employed language, regardless of the grammatical categories employed more. Based on the premise that Maltese children’s word production reflects the linguistic input received, including the contact phenomena and language choices made by native Maltese-English bilinguals, documentation of word usage also has implications for young Maltese children’s language milieu. For instance, this study’s finding of a consistent English language presence among children’s more commonly used words, accompanied by a substantial Maltese component, potentially reflects the relative salience of both languages in children’s input, while possibly also mirroring societal trends in the language choices incorporated in child-directed language use.

The commonality analyses reported here emerged as a valid and efficient means of uncovering trends in vocabulary acquisition. The more commonly produced words appeared to condense trends and trajectories in word production typically identified across children’s full range of expressive vocabularies. Specifically, the developmental reorganization of grammatical categories in the participants’ more commonly used words tended to replicate findings reported in larger studies of vocabulary acquisition. The more commonly produced words across children might be seen as a method-specific ‘core’ central to the acquisition of a particular language or language pair.

The merits of parental report are widely recognized and often seen to exceed its pitfalls. Parents’ observations of their children’s emergent language, across daily settings and with various interlocutors, enable them to report comprehensively on their children’s expressive vocabulary skills (Frank et al., 2021). When parents complete vocabulary checklists on the basis of their children’s daily word production, they not only provide researchers and clinicians with a reliable and valid estimate of their children’s language skills but they also contribute to a wider corpus that might be used for reference or norming purposes. Consideration of word usage adds depth to parent-based vocabulary measures. Examining how many children use specific checklist words sheds light on the relevance of these items in sensitively gauging various levels of vocabulary ability. In the study reported here, direct comparison between the more commonly reported words and those sampled naturalistically yielded objective indications as to which checklist words were more likely to resonate with reporting caregivers, compared to sampled commonality, and which items were well beyond the upper developmental limit of the target age range, as in the items that were never reported or sampled.

### 4.1. Limitations and recommendations

There are various limitations in this study that need to be acknowledged. Although typical development was a criterion for participant selection, it cannot be excluded that some participants may have been presenting with subtle language difficulties that were unidentified at the time of data collection. The small sample size, largely determined by the labor-intensive methodology, inevitably limited statistical power in analyses. Although additional dual-method data, collected in a longitudinal arm of this study, were available for few other 24-and 30-month-olds (two separate longitudinal cohorts, *N* = 9 and 7 respectively), their addition to the present cohort would have unbalanced the close similarity across age groups in numbers of participants and gender distribution. In retrospect, however, it would have been useful to top up the 30-month-group by one or even two participants, partly due to it being the smallest age group and also because this age point revealed an intense word usage dynamic that would have benefited from more extensive investigation. In addition, the computation of proportion scores for grammatical and language components would have enabled a more equitable comparison across methods than raw scores. Moreover, choice of the 50% + usage threshold was somewhat arbitrary, although partly influenced by Fenson et al.’s (1994) consideration of this level in their individual item analyses of the CDI norming dataset. Our intention was to pitch word-level analysis at a level that would not favor the more linguistically advanced participants. On the other hand, data at lower levels of usage, such as 25–49%, would have enabled more fine-grained insight on the levels of difficulty and psychometric sensitivity of a broader range of words. This is particularly relevant since the study of early language acquisition in Maltese children is still in its infancy, with no previous evidence documenting the specific words appearing earlier and later in typical development. Analysis of more commonly used words as a function of gender and vocabulary size level would have also added depth to the current findings but could be considered as a possible avenue for further research. For instance, Weber et al. (2018) employed item response models to investigate the probability of items on the newly-adapted Wolof version of the CDI vocabulary checklist eliciting responses in relation to their difficulty level, as well as child characteristics such as gender and level of vocabulary ability.

### 4.2. Conclusion

The present study hopes to contribute toward bridging a conspicuous gap in the research literature. It compares word-level measures obtained in parallel for the same children using two methods, parent report and language sampling, with a focus on an under-researched language pair. When analyzed as a function of age, the more commonly reported words were noticeably more numerous than those sampled, with a relatively limited number of item-level matches. Nonetheless, when the more commonly produced words identified through both methods were analyzed in terms of the grammatical categories and languages they represented, positive and significant correlations resulted. The shifting distributions of grammatical categories in the words more commonly sampled and reported were similar. Even more striking was the resemblance in language profiles documented at 18, 24 and 30 months by both methods. In spite of its unprecedented examination of word usage trends documented by caregiver report and language sampling employed in parallel, the present study has only scratched the veritable tip of the iceberg – it draws on a modest sample of children, with a focus restricted to the words used by 50% and over of participants at four age levels. While breaking into previously uncharted territory, this study clearly flags a need for research that investigates larger samples of children with a denser distribution of age points. The resource demands of transcription and analysis of language sample data inevitably limit the numbers of children from whom naturalistic data are collected. Nevertheless, it is still recommended that a sub-sample of children in any parent-report based study are assessed directly, not only to increase the robustness of the methodological design but also to add important insights based on researchers’ ‘lived experience’ in working with authentic and naturally-occurring data. Moreover, the present investigation showed sampled word usage to be well-honed in capturing shifting distributions of grammatical categories and languages despite its characteristically small numbers, further highlighting its methodological relevance.

An additional purpose of this study was to derive guidelines for objectively revising the Maltese-English CDI adaptation. The word commonality findings presented here can assist in prioritizing items to retain and eliminate from the current version, while prompting reflection on caregivers’ predisposition toward reporting nouns. Moreover, the current instrument’s language bias, deriving from it being originally adapted for children raised in Maltese-dominant homes, does not make it sufficiently comprehensive for English-dominant children and relatively balanced bilinguals in the Maltese childhood population. Striking a balance between Maltese and English components in the revised vocabulary checklist is called for.

Finally, the present findings can also inform functional vocabulary targets for young children with language difficulties. Although available research evidence related to the developmental trajectory of grammatical categories is relevant to speech and language intervention, word usage data add insight on the words that are likely to be employed in child-directed language and those likely to be picked up and internalized by young Maltese children. These words might well be relevant targets for children who are struggling with their acquisition of Maltese and English.

## Data availability statement

The original contributions presented in the study are included in the article/Supplementary material, further inquiries can be directed to the corresponding author.

## Ethics statement

This study involved human participants and was approved and reviewed by the University of Malta Research Ethics Committee. Written informed consent to participate in this study was provided by the participants’ legal guardian/next of kin.

## Author contributions

DG conceptualized and designed the study, wrote the first draft of the manuscript, and revised the manuscript. CG organized the database. LC performed the statistical analysis. LC and CG each wrote a section of the manuscript. All authors read and approved the submitted version.

## Funding

This work was partly supported by University of Malta Research Seed Grant 2023 (Ref. CMTRP01-23) awarded to DG.

## Conflict of interest

The authors declare that the research was conducted in the absence of any commercial or financial relationships that could be construed as a potential conflict of interest.

## Publisher’s note

All claims expressed in this article are solely those of the authors and do not necessarily represent those of their affiliated organizations, or those of the publisher, the editors and the reviewers. Any product that may be evaluated in this article, or claim that may be made by its manufacturer, is not guaranteed or endorsed by the publisher.
