# Supplementary material for: Word usage as measured by parental checklists and language samples: trends, comparisons, and implications
Source: Front Psychol. 2023 Aug 3;14:1214518. doi: 10.3389/fpsyg.2023.1214518 (PMC10434779; doi:10.3389/fpsyg.2023.1214518)
Supplement: Supplementary file 1 [file Table_1.DOCX]

Supplementary Material

Word usage as measured by parental checklists and language samples: trends, comparisons and implications

Daniela Gatt^1*^, Liberato Camilleri^2^, Chloe Grech^1^

^1^Department of Communication Therapy, Faculty of Heath Sciences, University of Malta, Msida, Malta

^2^Department of Statistics and Operations Research, Faculty of Science, University of Malta, Msida, Malta

***Correspondence:**Daniela Gatt Ph.D.
daniela.gatt@um.edu.mt

# Supplementary Tables

Table A. Reported words produced by 50% and over of 18-, 24- and 30-month-old children, in order of increasing commonality

| **Comm-onality** | **Age (months)** | | | |
| --- | --- | --- | --- | --- |
|  | 12 (*N* = 11) | 18 (*N* = 12) | 24 (*N* = 11) | 30 (*N* = 10) |
| 5 |  | -- | -- | Ajruplan  Balloon  Banni bannozzi  Basla  Bil-lejl  Bilqiegħda  Blow  Boots  Bott  Chicken nuggets  Ċikkulata  Clothes  Dance  Dawra  Dingdong  Dustbin  Fekruna  Fingers, toes  Frawli  Frott  Gallettina  Għand  Għatx  Ġins  Green  Gwardarobba  Ħalq  Ħelikopter  Ħwejjeg  Iebes, iebsa  Ingwanti  Kafe’  Kbir, kbira  Kelb  Key  Kitarra  Klawn  Knisja  Kollox  Kollu,Kollha  Kraker  Krema  Ktieb  Kuluri  Labar tal-inxir  Lady  Lanġasa  Liema  Lion  Lolipop  Ma’  Mela  Meta  Mxarrab, Mxarrba  Owkej  Pala  Paqpaq  Petne’  Piżelli  Pjanu  Police  Pool  Post fejn toqgħod  Purtiera  Qanpiena  Radio  Ritratt  Sandli  Seesaw  Silġ  Skola  Soap  Spider  Sponge  Swaba’  Tagħlaq  Taħt  Tal-ħobż  Tara  Tbus  Tħabbat  Tħobb  Tiekol  Tifel  Tiġieġa  Tistaħba  Tiżfen  Toilet paper  Torta  Train  Twaqqa’  Uff  Ukoll  Vapur  Wara  Wejfer  Wieħed, waħda  Work  Xampu’  Xemgħa  Xiex  Xugaman  Xxxx  Zalzett  Żebra  Żiemel  Zinn zinn zinn  Żokra  Żurżieqa |
| 6 | Aħħ  Nanna | Bejbi  Bumma  Car  Gażaża  Gowl  Kwakwak  Mimmi  Muuu  Pupa  Tlatlo  Vummvumm | Ajma  Baħar  Bandli  Bard  Basket  Beepbeep  Bus  Cheese  Chicken, hen  Clothes  ċuff  Dak, dik, dawk  Dar  Doctor  Ears  Flower  Friġġ  ħalib  Ieħor, oħra, oħrajn  Issuq  Kwakwak  Money  Mouth  Mutur  Nappy  Nice  Orange  Panty  Paper  Patata  Potty  Pupa  Siġġu  Socks  Sodda  Sun  Te’  Terfa’  Tibki  Tilbes  Tipparkja  Tixrob  Wash_ RECALLED  żaqq | Aeroplane  Ajma  Bear  Bitħa  Blu  Borma  Bozza  Bużżieqa  Chair  Cheese  Chicken, hen  Clap  Ċuff  Cupboard  Doctor  Dress  Duck  Fann  Fenek  Flixkun  Garaxx  Ġelat  Għada  Għandek  Għasfur  Ġuħ  Ħajt  Ħanut  Ħa, se  Ħaxix  Ħelu, ħelwa  Iċċapċap  Iddoqq  Id, idejn  Imħadda  Isfel  Issajjar  Ixxarrab  Kaxxa  Kexxun  Kwakwak  Larinġa  Lest, lesta  min  money  monkey  parti  Pencil  Pet’s name  Pink  Pupa  Ras  Rieqed, rieqda  Sheep  Sink  Snien  Story  Tapp  Teddy bear  Teeth  Terfa’  Tieħu  Tieqa  Tiġieġa  Tilgħab  Tispiċċa  Titlaq  Tkisser  Toqgħod  Trakk  Wash_ RECALLED  White  Wiċċ  Widnejn  Xadina  Xawer  Xejn  Xogħol  Żgħir, żgħira  Zokkor |
| 7 |  | Book  Koko’  Nannu  Pet’s name  Thank you | Banana  Boat  Braxx  Chicken  Chocolate  Festa  Fingers, toes  Għaġin  Hawn, hawnhekk  ħobża  Iva, eħe  Jiena  Lady  Mimmi  Mmm  Muuu  Naughty  Platt  Tmur  Tqum | Aħna  Arloġġ  Baħar  Bajda  Ballun  Bard  Barmil  Bebbuxu  Beepbeep  Bumma  Ċavetta  Cow  Daqshekk  Drum  DVD  Ears  Elephant  Festa  Fjura  Flower  Flus  Furketta  Ġebla  Għajnejn  Għaliex  Ġurdien  Hawn, hawnhekk  Horse  Iċċempel  Ice cream  Int  Issuq  Iżjed, iktar  Karozza  Karta  Kċina  Kejk  Ma...x  Mbagħad  Mera  Milk  Mouth  Pastizzi  Photo  Pig  Qattus  Ravjul  Riħ  Ross  Rota  Sapuna  Sieq, saqajn  Socks  Spagetti  Stro’  Sweets  Tadama  Taħsel  Taraġ  Tiftaħ  Tilbes  Tipparkja  Tixgħel  Tixtri  Toothbrush  Tpinġi  Tuffieħa  Tweġġa’  U  Wara  Wuwwuw  Xagħar  Xkupa  Yellow  żarbun  żebbuġ |
| 8 |  | Ammamm  Bahh  Ball  Piff jaqq  Mjaw  No | Aeroplane  Ammamm  Bajrow  Bejbi  Bieb  Biscuit  Bravu, brava  Bumm  Bumma  Cat  Dan, din, dawn  Dudu  Fish  Flus  Girl  Horse  Ilma  Kiss  Man  Nose  Please  School  Uff  Vummvumm  Wuwwuw  Xita  Xxxx | Art  Bajrow  Ball  Banju  Barmil  Basket  Biscuit  Bloks  Bravu, brava  Braxx  Brodu  Bumm  Bus  Butir  Buttuna  Child’s name  Christmas  Ċintorin  Ċipps  Colours  Dak, dik, dawk  Dan, din, dawn  Dawl  Dubbiena  Dudu  Flokk  Fuq  ġewwa  Girl  Ħafna  Ħalib  Ħaxix  Imqass  Issa  Iva, eħe  Jacket  Jiena  Juice  Kalzetti  Kiss  Kompjuter  Kuċċarina  Kuxin  Laħam  Maħmuġ, maħmuġa  Man  Mejda  Mjaw  Mmm  Mutur  Muuu  Nappy  Naughty  Ngħas  Nose  Nuċċali  Orrajt  Paper  Papra  Pastaż, pastaża  Piff jaqq  Rabbit  Sauce  School  Shoes  Siġġu  Papoċċ  Piġama  Sabiħ, sabiħa  Star  Sufan  Tajjeb, tajba  Tapit  Taqbeż  Tazza  Televixin  Tibża’  Tiegħek  Tiegħi  Tiġri  Tixju  Tixrob  Tmur  Toast  Toilet  Toy  Tqil, tqila  Tqum  Trid  Water  Xemx  Yes  żaqq |
| 9 | Papà, daddy | Aħħ | Aħħ  Baħħ  Bird  Boy  Child’s name  Dog  Fish  Happy birthday  Hello  Le  Milk  Mjaw  Mobile  Nannu  Pet’s name  Qalbi  Toy | Aħħ  Apple  Bandli  Bankina  Barra  Bejt  Beritta  Bird  Bottle  Car  Cat  Chicken  Chocolate  Dar  Fish  Friġġ  Ftit  ġobon  Hello  ħelu  ħobza  Jogurt  Krim  Le  Mimmi  Mobile  Nannu  Nice  Orange  Panty  Patata  Patata  Perzut  Pizza  Present  Puxċer  Qalbi  Qalziet  Sikkina  Sodda  Taqa’  Te’  Telefon  Tibki  Tiġi |
| 10 | Mamà, mummy | Bye, ciao, tatà  Bumm  One, two, three… | Bye, ciao, tatà  Ball  Book  Koko’  No  One, two, three…  Piff jaqq  Shoes  Taqa’  Thank you | Bye, ciao, tatà  Banana  Bejbi  Biċċa  Bieb  Boat  Book  Boy  Dog  Fejn  Fish  Għaġin  Happy birthday  Ilma  Koko’  Mamà, mummy  Nanna  No  One, two, three...  Papà, daddy  Pipi’  Platt  Please  Potty  Sausage  Thank you  Xita |
| 11 |  | Papà, daddy  Wuwwuw | Car  Mamà, mummy  Nanna  Papà, daddy  Pipi’ | -- |
| 12 |  | Mamà, mummy  Nanna | *--* | *--* |

Table B. Sampled words produced by 50% and over of 18-, 24- and 30-month-old children*, in order of increasing commonality

| **Comm-onality** | **Age (months)** | | |
| --- | --- | --- | --- |
|  | 18 (N = 12) | 24 (N = 11) | 30 (N = 10) |
| 5 | -- | -- | baby  blu  dog  għandek (have)  għax (because)  green  -lu  mela (so)  -ni, -ja (prn, 1 sg.)  tagħti (give)  tajjeb, tajba (good)  te’ (tea)  x’inhu (what)  yellow |
| 6 |  | Child’s name  dak, dik, dawk (that, those) | bħal (same as)  bi (with)  car  child’s name  elephant  fejn (where)  -hom (prn 3pl.)  iċċempel (call)  lil (to)  ma…x (not)  one, two, three…  tiġi (come)  tilgħab (play)  toy  x’, xiex (what) |
| 7 | dan, din, dawn (this, these) | hawn, hawnhekk (here)  baby | mamà, mummy  le (no)  papà, daddy  ieħor, oħra, oħrajn (another)  u (and)  -li (prn 1sg.)  tagħmel (do) |
| 8 | mamà, mummy |  | -ha (prn. 3f. sg.)  hekk (so)  ħa, se (fut. particle)  il-, l-… (def. art.)  jiena (I)  telefon  -u, -h (prn. 3m.sg.) |
| 9 |  |  | dak, dik, dawk (that, those)  hemm, hemmhekk (there)  iva, eħe (yes)  persons’ names  ta’ (of) |
| 10 |  | dan, din, dawn (this, these) | dan, din, dawn (this, these)  hawn, hawnhekk (here)  taqa’ (fall)  tara (see) |
| 11 |  |  | -- |
| 12 | -- | *--* | *--* |

*No words were sampled for 50% and over of 12-month-olds.
